# Room temperature-synthesized vertically aligned InSb nanowires: electrical transport and field emission characteristics

**DOI:** 10.1186/1556-276X-8-69

**Published:** 2013-02-11

**Authors:** Cheng-Hsiang Kuo, Jyh-Ming Wu, Su-Jien Lin

**Affiliations:** 1 Department of Materials Science and Engineering, National Tsing Hua University, No. 101, Sec. 2, Kuang-Fu Rd., Hsinchu 30013, Taiwan; 2 Department of Materials Science and Engineering, Feng Chia University, Taichung 40724, Taiwan

**Keywords:** InSb nanowires, Electrical transport, Field emission, Electron accumulation layer, Electrochemical method

## Abstract

Vertically aligned single-crystal InSb nanowires were synthesized via the electrochemical method at room temperature. The characteristics of Fourier transform infrared spectrum revealed that in the syntheses of InSb nanowires, energy bandgap shifts towards the short wavelength with the occurrence of an electron accumulation layer. The current–voltage curve, based on the metal–semiconductor–metal model, showed a high electron carrier concentration of 2.0 × 10^17^ cm^−3^ and a high electron mobility of 446.42 cm^2^ V^−1^ s^−1^. Additionally, the high carrier concentration of the InSb semiconductor with the surface accumulation layer induced a downward band bending effect that reduces the electron tunneling barrier. Consequently, the InSb nanowires exhibit significant field emission properties with an extremely low turn-on field of 1.84 V μm^−1^ and an estimative threshold field of 3.36 V μm^−1^.

## Background

Group III-V semiconductor nanowires, i.e., InAs, InP, GaAs, GaP, and InSb, have attracted substantial scientific and technological interests in nanoelectronic devices due to their high electronic transfer characteristic with low leakage currents. Meanwhile, the existence of an electron accumulation layer occurs near the material surface that causes high surface sensitivity and electric conductivity [1]. Among the III-V group, indium antimony (InSb) bulk (*E*_g_ = 0.17 eV, at 300 K) is a promising III-V direct-bandgap semiconductor material with zinc-blende (FCC) structure. Due to its narrow bandgap, InSb is extensively used in the fabrication of infrared optical detectors, infrared homing missile guidance systems, and infrared astronomy [2-4]. Next, a significant advantage of InSb is that it has extremely high electron mobility (electron mobility of 77,000 cm^2^ V^−1^ s^−1^) that resulted from the natural small effective mass (m* = 0.013 *m*_e_) and the ballistic length (up to 0.7 μm at 300 K), which are higher than those of any known semiconductor [5,6]. Hence, there is significant interest in InSb for the fundamental investigation of its nanostructure for potential application as nanoelectronic devices.

Interestingly, owing to their high surface-to-volume ratio and quantum confinement effect, one-dimensional (1-D) semiconductive nanostructures exhibit unique optical, electronic, and transport properties, which are widely applied in photoconductors [7], electron field emitters [8], and dye-sensitized solar cells [9]. In the middle of these various application fields, 1-D electron field emission has attracted wide attention recently due to the sufficient high current density obtained from small electrical field. It is because a cone nanostructure (usually several hundred nanometers) is able to greatly amplify the electrical field within an extremely tiny region of the tips. Nanostructures have consequently served as the proper candidates for electron field emitters [10].

Up to now, different thermal synthesis methods have been used to produce InSb nanowires, i.e., chemical beam epitaxy [11], chemical vapor deposition [12], and pulsed laser deposition [13]. However, the fast and simple synthesis of stoichiometric InSb nanostructures is also of priority concern. The different partial vapor pressures of In and Sb make it difficult to form the InSb compound. In particular, the low bonding energy of InSb causes the tendency of In and Sb to dissociate over 400°C. Additionally, the In-rich and Sb-rich regions derive from the large different melting points of In and Sb elements. Therefore, synthesizing InSb nanowires via thermal synthesis method is a challenging task since the growth of stoichiometric InSb nanowires requires precisely critical temperature control [6,12,14]. To address this concern, this work has utilized the electrochemical method at room temperature to fabricate single-crystal InSb nanowires with an anodic aluminum oxide (AAO) template. The synthesized process was a simple, fast, low-temperature (avoids the phase dissociation at a high temperature), and straightforward process for fabricating large-area, highly ordered, aligned InSb nanowires. Furthermore, the as-prepared InSb nanowires are expected to possess the electron accumulation layer on the surface. Importantly, the electron accumulation layer significantly affects the optical, transport, and field emission characteristics.

## Methods

**Figure 1:**
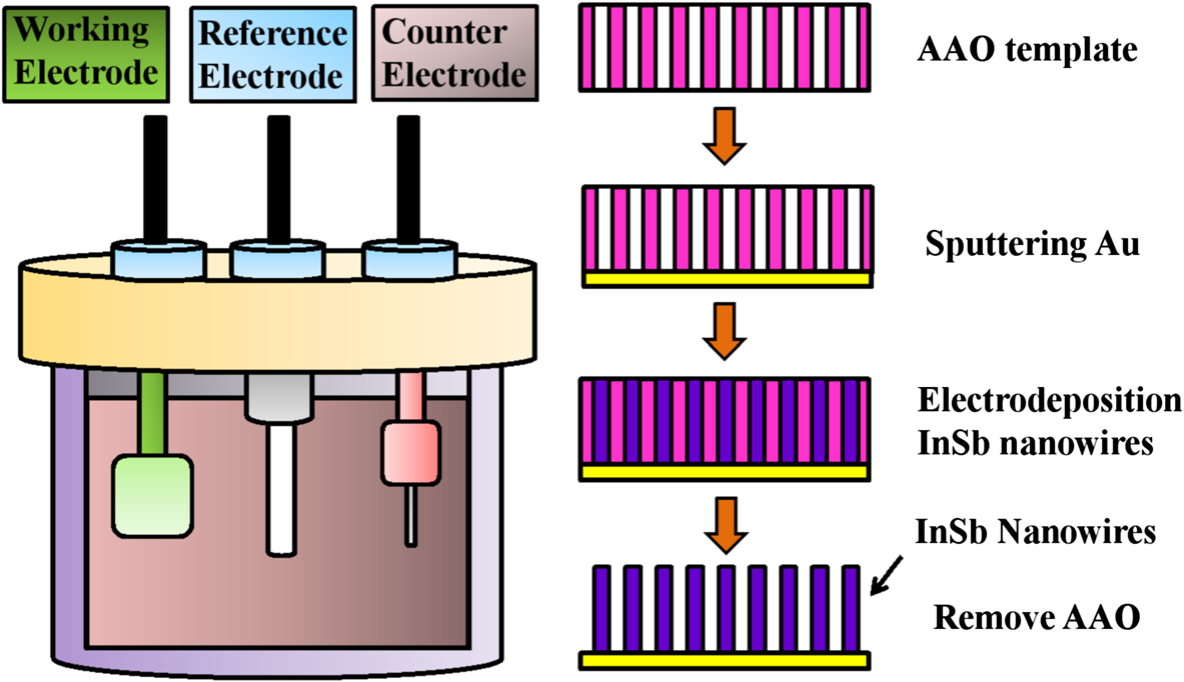
The schematic diagram of electrode position.

The fabrication of InSb nanowires is described as follows: The AAO template was purchased from Whatman® (GE Healthcare, Maidstone, UK). The diameters of the circular pores in the AAO were about 200 nm, and the thickness was about 60 μm. A gold (Au) film coated on the AAO template was used as the conductive layer for nanowire growth. The electrolyte was composed of 0.15 M InCl_3_, 0.1 M SbCl_3_, 0.36 M C_6_H_8_O_7_ · H_2_O, and 0.17 M KCl. The solvent of the electrolyte was distilled water. The InCl_3_ and SbCl_3_ provide metal ion source, and the C_6_H_8_O_7_ · H_2_O was utilized to allow the deposition potential of In and Sb to be close to each other. Figure 1 illustrates the schematic diagram of electrodeposition. The Au film on AAO was regarded as the working electrode. A platinum wire and Ag/AgCl electrode were applied as the counter electrode and reference electrode, respectively. The deposition time was controlled at 30 min under the deposition potential of −1.5 V versus the Ag/AgCl reference electrode at room temperature. After the deposition, the sample was washed with distilled water, and then a 5 wt.% NaOH solution was used to remove AAO. The sample was immersed in NaOH solution for 5 min, and subsequently, the residual NaOH solution was washed with distilled water. Finally, InSb nanowires were obtained.

These as-prepared nanowires were examined using a field emission scanning electron microscope (FESEM; HITACHI S-4800, operated at 10 kV, Chiyoda-ku, Japan), a desktop X-ray diffractometer (Bruker, D2 Phaser, Madison, WI, USA), a high-resolution transmission electron microscope (HRTEM; JEOL JEM-3000 F, operated at 300 kV, Akishima-shi, Japan) with an energy-dispersive X-ray spectrometer (EDX), and an X-ray photoelectron spectroscopy system (XPS, PerkinElmer model PHI600 system, Waltham, MA, USA). The optical properties were then examined from a Fourier transform infrared spectrometer (Bruker, Verpex 70 V). For the transport measurement, the synthesized InSb nanowires were dispersed onto a SiO_2_/Si substrate with pre-patterned Pt/Ti electrodes through a photolithograph, then through e-beam evaporation and the lift-off process, respectively. Subsequently, the focused ion beam was used to deposit Pt, which connects wires between Pt/Ti electrodes. Finally, the current–voltage (*I*-*V*) measurements were carried out using the Keithley 237 (Cleveland, OH, USA). The field emission current density versus applied field (*J*-*E*) measurements were performed in a vacuum chamber with a base pressure of about 6 × 10^−6^ Torr at room temperature. The inter-electrode gap (distance) between the anode and the cathode (InSb nanowires) was controlled using a preci-sion screw meter. The Keithley 237 high-voltage source-measurement unit was used to provide the sweeping electric field to record the corresponding emission currents.

## Results and discussion

**Figure 2:**
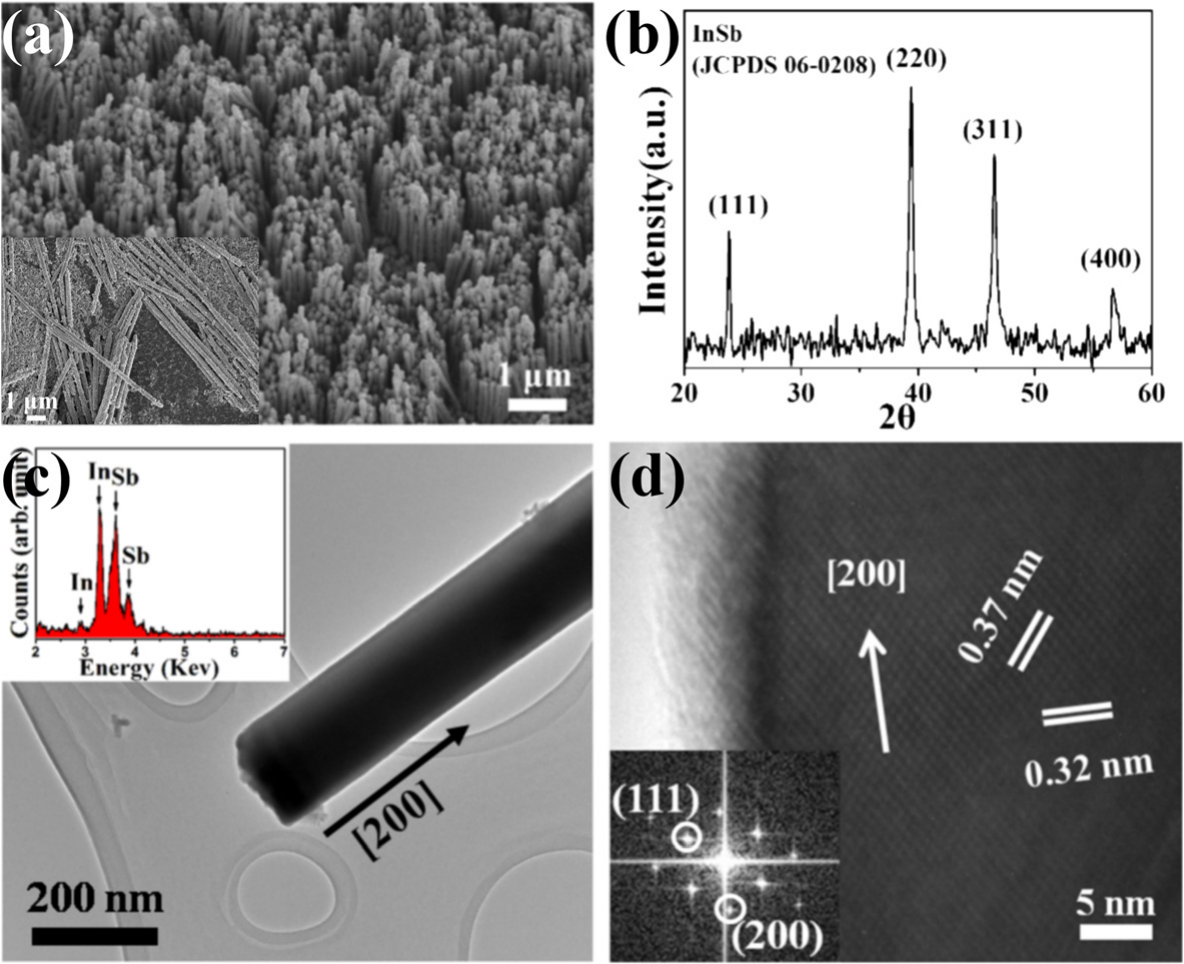
**SEM image, XRD pattern, and TEM and HRTEM images of the synthesized InSb nanowires.** (**a**) A SEM image showing the well-aligned, dense InSb. (**b**) XRD pattern of the synthesized InSb nanowires. (**c**) A TEM image of InSb nanowires revealing the preferred growth orientation being along [200], in which the image reveals the diameter (200 nm) of the InSb nanowires. Inset: EDX spectrum showing the composition of the synthesized InSb nanowire. (**d**) An enlarged HRTEM image showing the clear lattice spacings of atomic planes being about 0.37 and 0.32 nm. The inset is a FFT image.

The typical FESEM image seen in Figure 2a indicates that there are many InSb nanowires that they are well aligned and uniformly distributed on the Au layer and have diameters of around 200 nm, which corresponds to the pore size of AAO. The inset indicates that the length of InSb nanowires is about 5 μm. The as-prepared InSb nanowires have high aspect ratio. Figure 2b shows the XRD pattern that characterizes the zinc-blende structure of InSb (JCPDS 06–0208) with a lattice constant of 0.64 nm and, in addition, with no separate peaks of In and Sb. Next, in order to understand the morphology and crystalline nature of synthesized nanowires, the synthesized nanowires were characterized using TEM and HRTEM. Figure 2c depicts a TEM image of the synthesized InSb nanowire exhibiting a uniform width along its entire axis. The morphology is smooth and straight. The corresponding EDX spectrum in the inset of Figure 2c confirms that the element composition of the synthesized nanowire is only made of In and Sb, and the composition ratio of In/Sb is approximately 1:1. Figure 2d shows the HRTEM image of the InSb nanowire with the corresponding fast Fourier transform (FFT) as inset. Both the FFT pattern and the HRTEM image verify that the synthesized InSb nanowires have an excellent crystal quality with a preferred growth direction of [200]. The lattice spacings of 0.37 and 0.32 nm correspond to the (111) and (200) planes that could be indexed, which is consistent with an InSb zinc-blende phase.

**Figure 3:**
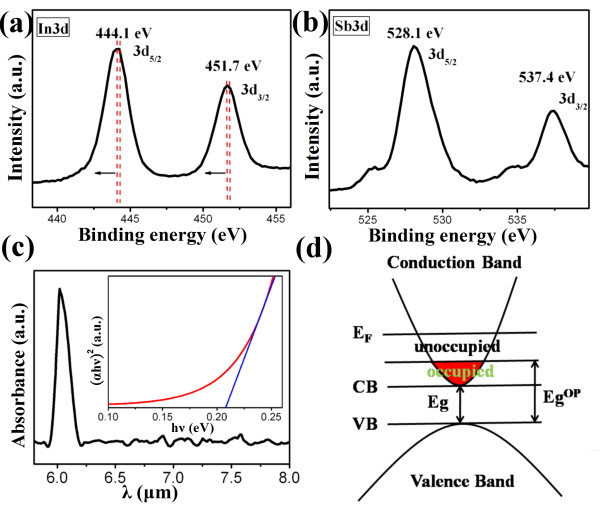
**XPS spectra of the synthesized nanowires.** (**a**) The In 3*d* core-level spectrum. (**b**) The Sb 3*d* core-level spectrum. (**c**) FTIR spectrum of the synthesized InSb nanowires. The inset shows (*αhν*)^2^ versus *hν* curve for InSb nanowires. (**d**) Schematic diagram of the InSb energy bandgap.

The surface states of the synthesized InSb nanowires were also investigated by pre-sputtering the specimen to remove surface contaminants before XPS analysis. The In 3*d* core-level spectrum indicated that the peaks of 444.1 eV (In 3*d*_5/2_) and 451.7 eV (In 3*d*_3/2_) correspond to the InSb species in Figure 3a. Figure 3b shows the Sb 3*d* core-level spectrum of the InSb nanowires. The Sb 3*d*_5/2_ and Sb 3*d*_3/2_ peaks refer to the InSb species at 528.1 and 537.4 eV, respectively [15,16]. Nevertheless, the In 3*d* peak experienced a downward shift of binding energy. A previous work observed the binding energy of the In 3*d* peak at 444.2 and 451.8 eV for bulk InSb [17]. Additionally, the In 3*d* peak shifted towards a low binding energy, which could be ascribed to the conversion in the bonding state of In ions due to the loss of Sb ions (Sb vacancies) in InSb nanowires. Therefore, the shielding effect of the valence electrons in In ions was increased due to a loss of the strong electronegativity of Sb that decreased the binding energy of the core electrons in In ions [18]. Moreover, InSb had a low binding energy of 1.57 eV, and Sb was easily vaporized due to a low vapor pressure temperature, subsequently leading to the formation of Sb vacancies [13,19,20]. The InSb are expected to have n-type semiconductivity that resulted from the anion vacancies [20-22]. The excess carrier may have originated from the Sb vacancies in InSb nanowires. A previous semiconductor-related work described the vacancy-induced high carrier concentration in 1-D nanoscale because the nanowires with a high surface-to-volume ratio easily led to more vacancies [23-26]. Moreover, previous works observed that the synthesized InSb nanowires indeed have a high electron concentration, which is about 3 orders of magnitude higher than those of bulk and thin films [13,14,19,27]. Accordingly, the InSb nanowires in this work may have high electron concentration.

Figure 3c shows the Fourier transform infrared (FTIR) spectral analysis of InSb nanowires. FTIR spectrum analysis of the InSb nanowires was undertaken to investigate the optical property in the wavelength in which the energy bandgap is located. A sharp rise in adsorbance occurs near 6.1 μm, which corresponds to the energy bandgap of 0.203 eV. The inset shows the (*αhν*)^2^ versus *hν* curve of the corresponding sample, where *α* is the absorbance, *h* is the Planck constant, and *ν* is the frequency. The absorption edges deduced from the linear part of the (*αhν*)^2^ versus *hν* curve allow an understanding of the energy bandgap for the InSb nanowire, which is about 0.208 eV and is consistent with the value obtained directly from the absorption spectrum. The energy bandgap of InSb increases only when the diameter is smaller than 65 nm. Once the diameter of InSb decreases to 30 nm, the energy bandgap will increase to 0.2 eV [28]. The diameter of the synthesized nanowires is 200 nm. Accordingly, the quantum confinement effect does not occur in this work. Here, the energy bandgap of InSb increased from 0.17 to 0.208 eV due to the high carrier concentration effect. Figure 3d schematically depicts the InSb energy bandgap. The increase in the energy bandgap was due to excess electrons filling up low-energy states in the conduction band. In other words, the excitation of electrons moved to a high-energy state (i.e., unfilled orbital) at the bottom of the conduction band (*E*_g_^op^). The excess electrons caused an enlargement of the energy bandgap, known as the Burstein-Moss (BM) effect [29-31]. The BM effect is an important phenomenon for n-type semiconductors. According to this theory, the Burstein-Moss shift (Δ*E*_BM_) depends on the electron concentration, as shown below [32]:

(1) $$\Delta E_{\mathrm{BM}}=\left(1+\frac{m_{e}^{*}}{m_{h}^{*}}\right)\left\{\frac{{\left(\frac{3}{\pi }\right)}^{\frac{2}{3}}h^{2}}{8m_{e}^{*}}n^{\frac{2}{3}}-4\mathrm{kT}\right\},$$

where *n* is the electron carrier concentration, *k* is the Boltzmann constant, and *T* is the absolute temperature. The *m*_e_^*^ and *m*_h_^*^ are the effective masses of electron and hole, respectively. Given that *m*_e_^*^ = 0.014 *m*_0_ and *m*_h_^*^ = 0.43 *m*_0_, the electron carrier concentration could be calculated from Equation 1. According to the calculation, the electron carrier concentration was 3.94 × 10^17^ cm^−3^, which is more than the intrinsic carrier concentration of InSb [2]. Therefore, the enlargement of energy bandgap and high electron density characteristics verified that the synthesized InSb nanowires are degenerate semiconductors, of which the Fermi level is located above the conduction band minimum [29]. Based on the theoretical calculation using Equation 1, during the crystal growth process, the high carrier concentration can be ascribed to the formation of Sb vacancies in InSb nanowires.

**Figure 4:**
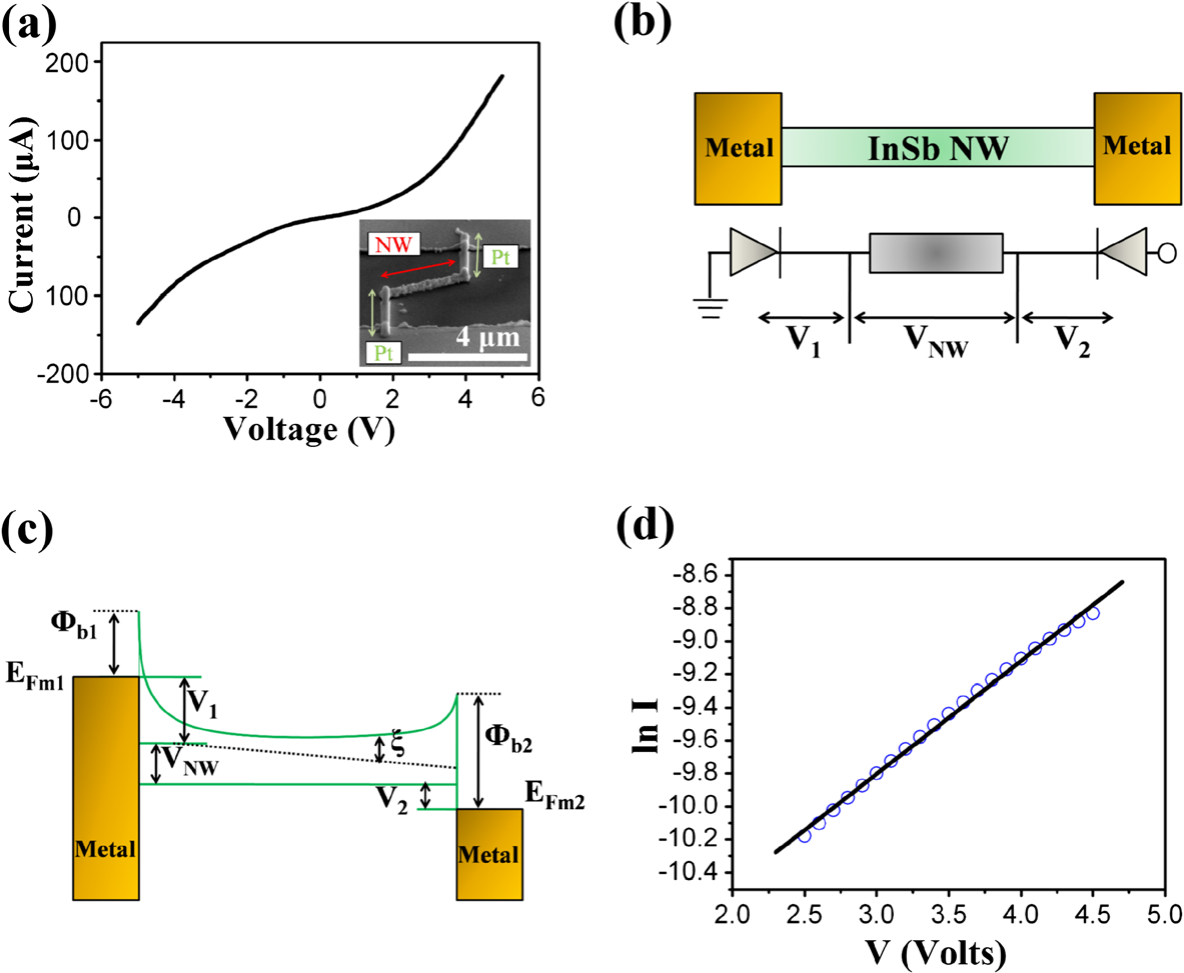
***I*-*V *curves and M-S-M structure and its energy band diagram.** (**a**) The almost symmetric *I*-*V* curve. The inset shows a representative FESEM image of InSb nanowire-based M-S-M structure. (**b**) Schematic diagram of the M-S-M structure and its equivalent circuit. (**c**) Energy band diagram of the M-S-M structure under applied voltage *V*. *Φ*_b1_ and *Φ*_b2_ are the corresponding Schottky barrier heights at the two contacts, and *ζ* is the distance between the bottom of the conductance band and the *E*_F_ of the InSb nanowire. (**d**) The *I*-*V* curve of ln (*I*) versus *V* for InSb nanowire.

To understand the transport characteristics of InSb nanowires, a single InSb nanowire was connected with Pt electrodes to fabricate a nanodevice and measured using a high-power electrical measurement system (Keithley 237), as illustrated in Figure 4a. The *I*-*V* curve shows the back-to-back Schottky contacts formed in between the Pt electrode and an InSb nanowire. The metal–semiconductor–metal (M-S-M) model for quantitative analysis of *I*-*V* characteristics of an InSb nanowire was applied to fit the variables. Based on this M-S-M model, one can estimate the intrinsic parameters of the InSb nanowire. Figure 4b schematically depicts the semiconductor nanowire-based M-S-M structure and its equivalent circuit. Figure 4c shows the energy band diagram of the M-S-M structure. The voltages on barrier 1, the nanowire, and barrier 2 are denoted as *V*_1_, *V*_NW_, and *V*_2_, respectively. This provides the following equation:

(2) $$V=V_{1}+V_{\mathrm{NW}}+V_{2}.$$

At low bias (<0.1 V), the *V* is distributed mainly on the two Schottky barriers (*V*_1_, *V*_2_ ≫ *V*_NW_). Particularly, the voltage drop on the reverse-biased Schottky barrier 1 increases rapidly and becomes dominant until about 2 V when the current becomes notable. At the same time, *V*_NW_ becomes non-negligible. Furthermore, the voltage drop across the forward-biased Schottky barrier 2 remains small. In the intermediate bias, the reverse-biased Schottky barrier dominates the total current *I*. Consequently, the total current *I* can be described as follows [33]:

(3) $$\text{ln}I=\text{ln}\left(\mathrm{SJ}\right)=\text{ln}S+V\left(\frac{q}{\mathrm{kT}}-\frac{1}{E_{0}}\right)+\text{ln}J_{S},$$

where *J* is the current density through the Schottky barrier, *S* is the contact area associated with this barrier, *E*_0_ is a parameter that depends on the carrier density, and *J*_S_ is a slowly varying function of applied bias. The logarithmic plot of the current *I* versus the bias *V* gives approximately a straight line of the slope *q*/*kT −* 1/*E*_0_, as shown in Figure 4d. The electron concentration *n* can be obtained by the following equations [34]:

(4) $$E_{0}=E_{00}\cot h\left(\frac{E_{00}}{\mathrm{kT}}\right),$$

(5) $$E_{00}=\frac{h}{4\pi }{\left(\frac{N_{d}}{m_{e}^{*}\varepsilon_{s}\varepsilon_{o}}\right)}^{\frac{1}{2}},$$

where *E*_00_ is an important parameter in tunneling theory, *N*_d_ is the electron concentration, *ε*_s_ and *ε*_0_ are the relative permittivity of the semiconducting nanowire and free space, respectively. As is estimated, the electron carrier concentration was 2.0 × 10^17^ cm^−3^, which is close to the estimative value of the BM effect. At the large bias, differentiating the *I*-*V* curve can obtain the total resistance associated with the nanowire. The resistivity *ρ* of 0.07 Ω cm was obtained from the *I*-*V* curve at large bias. Furthermore, according to *σ* = *nqμ*, the corresponding electron mobility *μ* of the InSb nanowire was estimated to be 446.42 cm^2^ V^−1^ s^−1^. The value is three times higher than that of reported n-type InSb nanowires [13]. However, the value is much smaller than those of the bulk and thin films. The reason of decay is attributed to the enhanced surface roughness scattering [13,35,36]. The nanowire surface becomes rough due to the presence of surface defects. Moreover, surface roughness scattering becomes strong and further limits the movement of electrons due to the decrease of nanowire diameter. It is still higher than that of known oxide semiconductor nanowires [33,37,38]. This implies that it has high potential for application in high-speed nanoelectronic devices.

**Figure 5:**
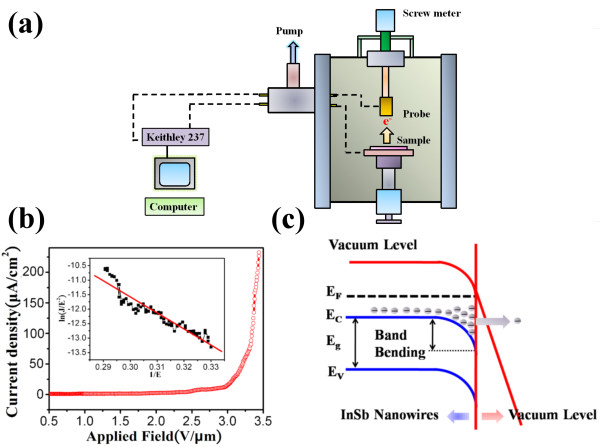
**Field emission measurement system, *J*-*E *field emission curve, and surface band diagram of InSb nanowires.** (**a**) The schematic diagram of field emission measurement system. (**b**) *J*-*E* field emission curve. The turn-on field of InSb nanowires is 1.84 V μm^−1^ at 1 μA cm^−2^, and the threshold field of InSb nanowires is 3.36 V μm^−1^ at 0.1 μA cm^−2^. Inset: F-N plot reveals the field emission behavior that follows F-N theory. (**c**) Schematic of the surface band diagram of the InSb nanowires.

In order to realize the potential applications of vertically aligned InSb nanowires in the area of nanoelectronics, electron field emission characteristics are analyzed based on the Fowler-Nordheim (F-N) theory. The uniform length, high conductivity, and high surface-to-volume ratio of vertically aligned InSb nanowires have strong local field enhancement factors that boost electron tunneling into a vacuum, so the vertically aligned InSb nanowires have excellent potential nanostructures for field emitters. Figure 5a illustrates the field emission measurement system. The field emission measurements were performed in a vacuum chamber with a base pressure of about 6 × 10^−6^ Torr at room temperature. The inter-electrode distance between the probe and the sample was controlled using a precision screw meter. The Keithley 237 high-voltage source-measurement unit was used to provide the sweeping electric field to record the corresponding emission currents. Figure 5b shows the electric field emission performance of InSb nanowires and describes the field emission current density dependence on applied electric fields. The field emission properties can be analyzed by the F-N theory [39] as is listed below:

(6) $$J=\left(A\beta^{2}E^{2}/\Phi \right)\exp\left(-B\Phi^{\frac{3}{2}}/\mathrm{\beta E}\right),$$

where *E* (*E* = *V*/*d*) expresses the applied electric field, *V* represents the applied voltage, *Φ* is the work function of the material, *β* is the field enhancement factor, and *A* and *B* are constants, where *A* = 1.56 × 10^−10^ (A V^−2^ eV) and *B* = 6.83 × 10^3^ (eV^−3/2^ V m^−1^) [39]. In previous works, the turn-on field defines the current density of 1 μA cm^−2^[39]. The turn-on field (*E*_on_) of InSb nanowires in this work is therefore 1.84 V μm^−1^. The obtained *E*_on_ value of InSb nanowires is excellent compared to the value of other reported materials via the thermal reactive process, such as SnO_2_/Sb nanowires (4.9 V μm^−1^) [40], SiC nanowires (5 V μm^−1^) [41], carbon nanotubes (4 V μm^−1^) [42], and AlN nanotips (3.9 V μm^−1^) [43]. Additionally, in order to generate enough brightness (>1,000 cd m^−2^) for an electronic device (i.e., display) under practical operation, the current density shall reach 0.1 mA cm^−2^[39]. Thus, the threshold field (*E*_th_) of InSb nanowires is around 3.36 V μm^−1^, so the generated current density can achieve enough brightness. Compared to the above-described materials via the thermal reactive process, this work synthesized InSb nanowires that not only exhibited excellent characteristics but also provided the advantages of room-temperature synthesis and a large area without expensive vacuum equipment.

The F-N emission behavior can be observed by plotting the ln(*J*/*E*^2^) versus 1/*E* curve, shown in the inset of Figure 5b. The linear curve implies that the field emission behavior of nanowires follows the F-N theory. Based on the F-N theory, the field enhancement factor *β* of InSb nanowires can be calculated. According to the work function of InSb (4.57 eV) [44], the field enhancement factor *β* is regarded as 20,300. Generally, the field emission performance is usually associated with the crystal geometry, the dimension of the material, emission height, crystal structure, conductivity, work function, and the density of nanostructures [45]. In this work, the excellent turn-on field (*E*_on_) of InSb nanowires can be attributed as follows: The high carrier concentration of the InSb nanowires with the Fermi level is located above the conduction band minimum, significantly reducing the effective electron tunneling barrier. Figure 5c illustrates the band diagram of degenerate InSb nanowires. The large density of states in the InSb conduction band (i.e., surface accumulation layer) causes a downward band bending near the surface region that eventually leads to lower the electron tunneling barriers. Additionally, the Fermi level is located above the conduction band minimum that can also improve the efficiency of tunneling at a low electric field. Next, the vertically aligned nanowires also play an important role. The high aspect ratio of the nanowires at applied electric field easily makes the electrons to accumulate on the surface and enhance significant field emission property. However, the density of nanowires must be moderate [46,47]. Previous works reported that the electrostatic screening effect increased the turn-on field and decreased the overall emission current density of densely packed grown nanowires [48,49]. This is because the applied electric field will overlap with that of the others. Consequently, the effective electric field of densely packed nanowires will be lowered compared to the stand-alone nanowires. Here, there is a reduced screening effect in the vertically aligned InSb nanowires due to a sufficient spacing between the emitters; meanwhile, there is the nanodimension structure with high aspect ratio. Therefore, the electron accumulation that occurs in the conduction band and sufficient spacing in aligned nanostructures can simultaneously enhance field emission property.

## Conclusions

Single-crystalline InSb nanowires can be successfully synthesized via the electrochemical method at room temperature. The *I*-*V* curve of the InSb nanowires based on the M-S-M model shows low resistivity *ρ* of 0.07 Ω cm owing to the existence of Sb vacancies. Meanwhile, InSb nanowires have a high electron concentration of 2.0 × 10^17^ cm^−3^ and a high electron mobility of 446.42 cm^2^ V^−1^ s^−1^. Also, the energy bandgap increases from 0.17 to 0.208 eV due to the filling up of low-energy states in the conduction band by excess electrons. Thus, the enlargement of energy bandgap and high electron concentration reveal that the InSb nanowires are degenerate semiconductors with the Fermi level located above the conduction band minimum. The accumulation layer occurs at the surface of InSb nanowires. The surface accumulation layer in the InSb conduction band causes a downward band bending near the surface region that eventually leads to lowering of the electron tunneling barriers. Moreover, a sufficient spacing between the InSb nanoemitter can significantly reduce the screening effect. Consequently, the vertically aligned InSb nanowires exhibit an extremely low turn-on field of 1.84 V μm^−1^ and an estimative threshold field at 3.36 V μm^−1^ when the current density was 1 μA cm^−2^ and 0.1 mA cm^−2^, respectively. The outstanding characteristics of InSb nanowires are highly promising for use in nanoelectronics, especially in the front area of flat panel displays and high-speed-response field-effect transistors.

## Competing interests

The authors declare that they have no competing interests.

## Authors’ contributions

CHK wrote the manuscript and performed all the experiments and the data analysis. SJL and JMW provided the information and organized the final version of the paper. All authors read and approved the final manuscript.
